# A Systematic Review of Herbal Medicines in the Management of Diabetes: Efficacy, Toxicological Profiles, and Clinical Safety Considerations

**DOI:** 10.7759/cureus.107618

**Published:** 2026-04-23

**Authors:** Amit Nampalliwar, Nilabh K Singh, Mohit Kher, Prashant U Sasane, Chandreshwar P Sinha, Umesh K Dixit

**Affiliations:** 1 Department Roga Nidan Evum Vikriti Vigyan (Diagnostics and Pathology), Government Ayurved College and Hospital, Bilaspur, IND; 2 Department of Medicine, Rajendra Institute of Medical Sciences and Hospital, Ranchi, IND; 3 Department of Pharmacology, Kanta Devi Medical College, Hospital, and Research Centre, Mathura, IND; 4 Department of Kayachikitsa (Internal Medicine), All India Institute of Ayurveda, Goa, IND; 5 Department of Kayachikitsa (Internal Medicine), Shri Narayan Prasad Awasthi (NPA) Government Ayurved College and Hospital, Pt. Deendayal Upadhyay Memorial Health Science and Ayush University of Chhattisgarh, Raipur, IND; 6 Department of Biochemistry, Sri Siddhi Vinayak Medical College, Sambhal, IND

**Keywords:** diabetes mellitus, glycaemic control, herbal medicine, metabolic safety, prediabetes

## Abstract

Diabetes mellitus and prediabetes represent major global health challenges associated with metabolic and cardiovascular complications. This review was conducted to evaluate herbal medicines as complementary strategies for glycaemic control and metabolic risk reduction. This systematic review assessed the efficacy, metabolic and cardiovascular effects, and clinical safety of herbal medicines used in diabetes and prediabetes management. A systematic literature search was conducted following Preferred Reporting Items for Systematic reviews and Meta-Analyses (PRISMA) 2020 guidelines. Eligible studies included clinical and relevant preclinical investigations reporting glycaemic, metabolic, cardiovascular, and safety outcomes of herbal interventions. Data were extracted descriptively and synthesized qualitatively due to heterogeneity in study design and outcomes. Eleven studies met the inclusion criteria. Most interventions demonstrated reductions in fasting blood glucose, postprandial glucose, oral glucose tolerance test values, and, in several cases, glycated hemoglobin. Improvements in lipid profiles and vascular parameters were also reported. Safety assessments revealed favorable tolerability, with no serious adverse events and stable hepatic and renal markers at studied doses. Findings from the included studies indicate that selected herbal medicines demonstrated adjunctive benefits in glycaemic control and metabolic parameters in diabetes and prediabetes. Further well-designed trials are needed to confirm long-term efficacy, safety, and clinical applicability.

## Introduction and background

Diabetes mellitus is a persistent metabolic condition characterized by chronic hyperglycemia resulting from defects in insulin secretion, insulin action, or both [1]. Over the past few decades, the prevalence of diabetes and prediabetes globally has risen considerably, posing a significant public health challenge [2]. Loss of glucose homeostasis leads to microvascular and macrovascular complications, including nephropathy, neuropathy, retinopathy, cardiovascular disease, and stroke [3]. Prediabetes is defined as an intermediate metabolic state characterized by impaired fasting glucose and/or impaired glucose tolerance, associated with a high risk of progression to diabetes and vascular dysfunction [4,5]. Pharmacological management remains the cornerstone of diabetes care and is effective in controlling hyperglycemia and reducing complications [6]. However, long-term use of conventional antidiabetic agents has been associated with adverse effects, reduced tolerance, and diminished efficacy in certain populations [7,8]. These limitations have been documented in previous pharmacological and clinical evaluations of antidiabetic therapies [9]. Consequently, there has been increasing interest in complementary approaches that may improve metabolic regulation and treatment acceptability [8]. Herbal medicines have long been used in traditional medical systems for metabolic disorders and continue to attract attention as adjunctive therapeutic options [10,11].

Evidence from both clinical studies (involving human participants) and preclinical studies (including in vivo and in vitro experimental models) indicates that several herbal medicines exhibit antihyperglycaemic effects through multiple biological mechanisms [12,13]. These mechanisms include improved insulin sensitivity, increased peripheral glucose uptake, reduced intestinal glucose absorption, enhanced insulin secretion, decreased oxidative stress, and modulation of lipid metabolism [14,15]. Herbal agents such as curcumin, cinnamon, *Aloe vera*, bitter melon, ginseng, berberine-containing formulations, and aged garlic extract have been evaluated in clinical studies involving individuals with diabetes or prediabetes [16,17]. In addition to glycaemic control, some interventions have demonstrated improvements in lipid profiles, vascular function, and cardiovascular risk markers, suggesting broader cardiometabolic benefits [18]. However, the available evidence is heterogeneous, limiting direct comparison across studies.

There is considerable variability across studies in population characteristics, intervention composition, dosage, duration, and outcome measures. Several prior studies and reviews have reported a predominant focus on glycaemic endpoints, with comparatively limited evaluation of metabolic, cardiovascular, and toxicological outcomes [19,20]. Similarly, inconsistencies in the reporting of adverse events, biochemical safety markers, and long-term tolerability have been highlighted in earlier literature [21,22], which limits confidence in clinical applicability. These gaps are particularly relevant given the increasing use of herbal medicines among individuals with diabetes and prediabetes.

Previous reviews on herbal interventions for diabetes have largely adopted narrative approaches, with limited parameter-specific or outcome-integrated analyses [23-25]. Comprehensive systematic evaluations that concurrently assess glycaemic efficacy alongside metabolic, cardiovascular, and safety outcomes remain limited [26,27]. Furthermore, methodological quality assessment and risk of bias evaluation are not consistently incorporated, reducing the robustness of conclusions. This lack of integrated synthesis of therapeutic efficacy and safety represents a key limitation in the current evidence base. Prediabetes warrants particular attention, as it is associated with early endothelial dysfunction, low-grade inflammation, and elevated cardiovascular risk even in the absence of overt diabetes [28]. Interventions that improve glucose regulation at this stage may delay disease progression and reduce long-term complications. Comparative evaluation of herbal medicines in both prediabetic and diabetic populations may therefore provide important insights into preventive and therapeutic roles. A systematic synthesis integrating glycaemic, metabolic, cardiovascular, and safety outcomes is essential to better define the clinical relevance of these interventions and to inform future research and evidence-based practice.

Objectives of the review

The objective of this systematic review is to evaluate the efficacy, metabolic and cardiovascular effects, and clinical safety of herbal medicines used in the management of diabetes and prediabetes. This review synthesizes available evidence to identify consistent outcomes and highlight gaps in safety reporting and methodological quality.

## Review

Methodology

Search Strategy and Data Sources

A systematic literature search was conducted to identify studies evaluating the efficacy, toxicological profiles, and clinical safety of herbal medicines in the management of diabetes and prediabetes. The following electronic databases were searched: PubMed/MEDLINE, Scopus, Cochrane CENTRAL, and Embase. The literature search was conducted from database inception to March 2026, and the final search was performed on 20 March 2026. Searches were performed using combinations of keywords including "diabetes", "prediabetes", "herbal medicine", "phytotherapy", "efficacy", "safety", and "toxicity", combined using Boolean operators (AND, OR). Controlled vocabulary (e.g., Medical Subject Headings (MeSH) terms in PubMed) was applied where appropriate, and the search strategy developed in PubMed was adapted for use in other databases. Only studies published in English were included. Additional studies were identified through manual screening of reference lists. The study selection process followed Preferred Reporting Items for Systematic reviews and Meta-Analyses (PRISMA) 2020 guidelines. Database-specific counts were not retained after deduplication.

An example of the PubMed search strategy was as follows: ("diabetes mellitus" OR "prediabetes") AND ("herbal medicine" OR "phytotherapy" OR "plant extract") AND ("efficacy" OR "safety" OR "toxicity").

Eligibility Criteria

Inclusion criteria: Studies were included if they evaluated herbal medicines or formulations in the management of diabetes mellitus or prediabetes and reported outcomes related to glycaemic control, metabolic parameters, safety, or toxicity. Eligible study designs included randomized controlled trials and controlled clinical trials. Studies were required to report dosage, duration, and outcome measures to ensure interpretability.

Exclusion criteria: Studies were excluded if they did not meet predefined population or intervention criteria, lacked outcome data, were non-English publications, or were reviews, editorials, conference abstracts, or exclusively in vitro studies without clinical or in vivo relevance.

Data Extraction and Synthesis

Data were extracted using a standardized process. Data extraction was performed independently by two reviewers using a predefined extraction form. Extracted variables included study citation, herbal intervention, study design, population, dosage, duration, efficacy outcomes, and safety findings. Data were summarised descriptively and presented in tabular form.

A qualitative synthesis was conducted due to heterogeneity in study design, interventions, and outcomes. A meta-analysis was not performed due to substantial heterogeneity in study designs, intervention types, dosages, durations, and outcome measures, which precluded meaningful statistical pooling of results. Findings were compared narratively to identify patterns of efficacy and safety.

Variability in reporting formats and the absence of consistent effect size measures, p-values, and confidence intervals across studies further limited the feasibility of quantitative synthesis. Therefore, a descriptive qualitative approach was adopted to summarise trends in efficacy and safety outcomes.

Where numerical data were available, representative mean values were extracted for graphical visualization. No meta-analysis was performed due to heterogeneity.

Quality Assessment

The methodological quality of included studies was evaluated based on study objectives, design, sample size, intervention details, and completeness of outcome reporting. Studies with clear methodologies, appropriate control groups, and transparent reporting were considered higher quality. Ethical reporting and adequacy of intervention protocols were also assessed.

Risk of Bias Assessment

Risk of bias was assessed using the Cochrane Risk of Bias (RoB 2) tool for randomized controlled trials, with an adapted approach for non-randomized studies. Domains assessed included randomization, allocation concealment, blinding, completeness of outcome data, and selective reporting.

Two reviewers independently assessed each study, and discrepancies were resolved by consensus. Predefined criteria were used for the classification of bias domains. Studies with robust randomization, blinding, and clear outcome reporting were considered low risk. Potential biases included small sample sizes, short durations, and inadequate reporting of adverse events. These limitations were considered during the interpretation of findings.

Results

Search Results

A systematic search identified records from multiple electronic databases. After removal of duplicates, titles and abstracts were screened for relevance, followed by full-text assessment of potentially eligible studies. Studies not meeting the inclusion criteria, lacking relevant outcome data, or published in non-English languages were excluded.

**Figure 1:**
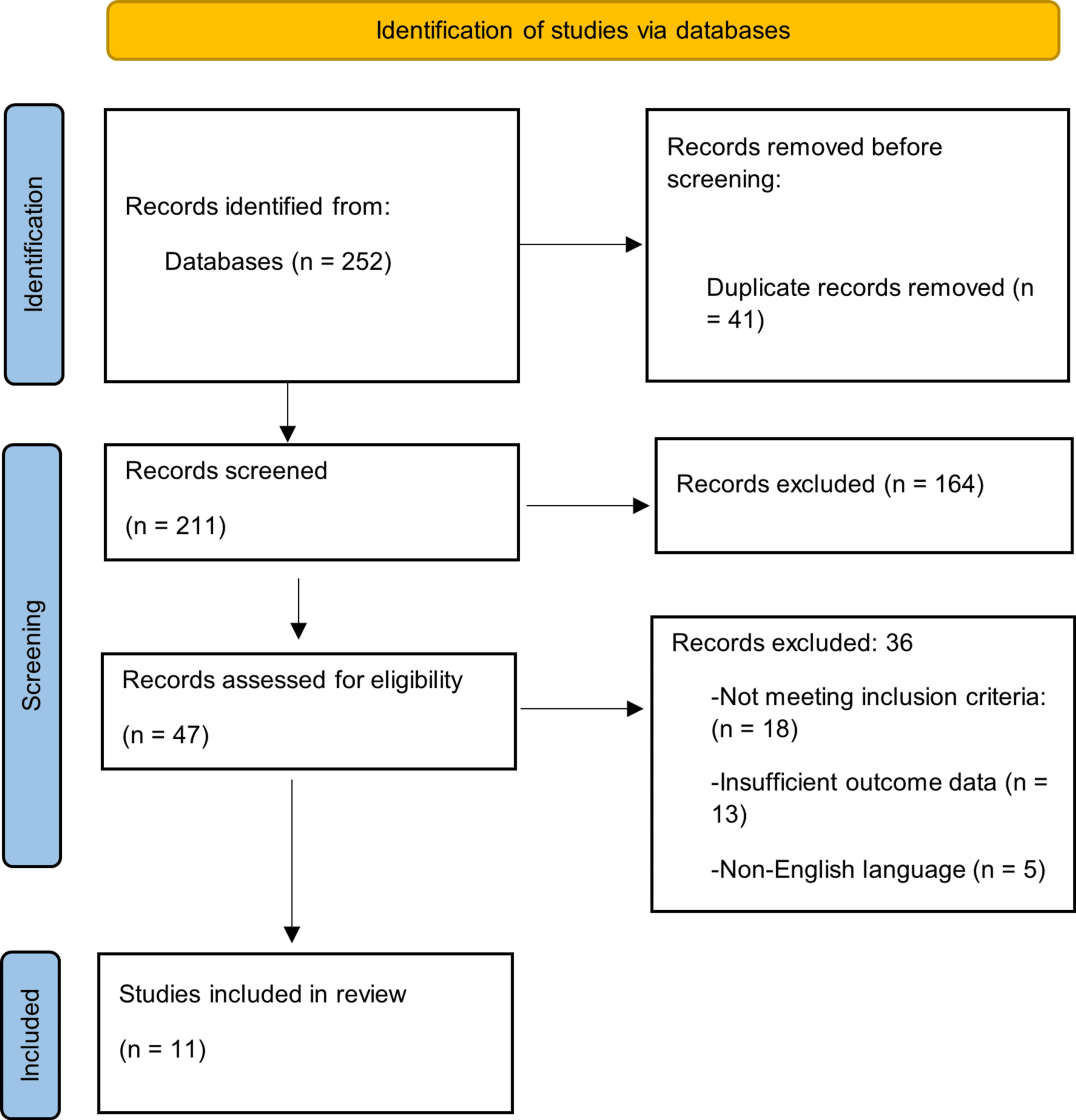
PRISMA flow diagram PRISMA - Preferred Reporting Items for Systematic reviews and Meta-Analyses

A total of 252 records were identified, of which 41 duplicates were removed, leaving 211 records for screening. After screening, 47 full-text articles were assessed for eligibility, and 11 studies were included in the final qualitative synthesis. The study selection process is presented in the PRISMA 2020 flow diagram (Figure 1).

Study Characteristics

The 11 included studies comprised randomized controlled trials, double-blind placebo-controlled trials, and active-controlled clinical trials. Study populations included individuals with type 2 diabetes mellitus, prediabetes, and those with metabolic risk factors. These populations reflect both disease-stage and risk-based cohorts relevant to diabetes progression.

Herbal interventions included both single agents, such as curcumin, cinnamon, *Aloe vera*, bitter melon, aged garlic extract, American ginseng, and Salvia miltiorrhiza, and multi-herbal formulations, including GlycaCare-II, HIMABERB® 2, and other polyherbal preparations.

The duration of interventions ranged from eight weeks to 12 months, with dosages varying across studies. This variability reflects differences in study design and intervention protocols. Table 1 summarises study characteristics, dosage regimens, glycaemic outcomes, and safety findings of the evaluated herbal medicines.

**Table 1:** Clinical and preclinical evidence of herbal medicines in diabetes management ALT - alanine aminotransferase; AUC - area under the curve; BID - twice daily; CAVI - Cardio-Ankle Vascular Index; FPG - fasting plasma glucose; GI - gastrointestinal; HbA1c - glycated hemoglobin; HOMA-IR - homeostatic model assessment of insulin resistance; HOMA-B - homeostatic model assessment of β-cell function; OGTT - oral glucose tolerance test; RCT - randomized controlled trial; TC - total cholesterol; LDL-C - low-density lipoprotein cholesterol; HDL-C - high-density lipoprotein cholesterol; TG - triglyceride

Study citation	Herbal medicine	Study design & population	Dose & duration	Key efficacy outcomes	Toxicological/clinical safety findings
Alinejad-Mofrad et al., [17]	Aloe vera	Double-blind randomised controlled trial; individuals with prediabetes	300 mg or 500 mg twice daily; 8 weeks	300 mg dose reduced fasting blood glucose and HbA1c; 500 mg dose improved lipid profile (↓TC, ↓LDL-C, ↑HDL-C)	No toxicity or serious adverse events reported
Romeo et al., [18]	Cinnamon	Double-blind, placebo-controlled, randomized trial; adults with prediabetes	500 mg three times daily; 12 weeks	Significant reductions in fasting plasma glucose, 2-h OGTT glucose, and glucose AUC compared with placebo	No serious adverse events; adverse-event frequency comparable to placebo; no clinically relevant laboratory abnormalities
Vuksan et al., [19]	American ginseng extract	Randomized, placebo-controlled add-on trial; patients with type 2 diabetes mellitus	1 g per meal (3 g/day); 8 weeks	Significant reductions in HbA1c and fasting blood glucose compared with placebo	Liver (ALT) and renal (creatinine) parameters unchanged; no hepatic or renal toxicity
Panigrahi and Mohanty, [20]	Berberine + silymarin (HIMABERB®)	Double-blind randomized controlled trial; individuals with prediabetes	Berberine 200 mg + silymarin 105 mg twice daily; 6 months	Significant reductions in fasting plasma glucose and 2-h OGTT glucose; higher reversion to normoglycemia	Well tolerated; no major safety concerns reported
Hamal et al., [21]	Aged garlic extract	Randomized, double-blind, placebo-controlled trial; patients with type 2 diabetes mellitus	2400 mg/day; 3 months	Significant improvement in cardio-ankle vascular index, indicating improved endothelial function	No significant hepatic, renal, or hematological toxicity
Khalili et al., [22]	Polyherbal formulation (silymarin + nettle + olibanum)	Randomized, double-blind, placebo-controlled trial; patients with type 2 diabetes mellitus	600 mg/day; 90 days	Significant reductions in fasting blood glucose and HbA1c; triglycerides decreased	No adverse events; liver and kidney function tests remained normal
Majeed et al., [23]	GlycaCare-II (multi-herbal formulation)	Randomized, double-blind, active-controlled trial; prediabetic and newly diagnosed type 2 diabetes patients	522.5 mg twice daily; 120 days	Significant reductions in HbA1c, fasting blood sugar, and postprandial blood sugar; efficacy comparable to metformin	No adverse events; liver, renal, and hematological parameters remained within normal ranges
Kim et al., [24]	*Momordica charantia *(bitter melon extract)	Randomized, placebo-controlled clinical trial; adults with prediabetes	2.4 g/day; 12 weeks	Significant reductions in OGTT glucose at 30 min and 120 min; decreased postprandial glucagon levels	No serious adverse events; liver enzymes and creatinine remained within safe limits
Panahi et al., [25]	Curcuminoids + piperine	Randomized, double-blind, placebo-controlled trial; patients with type 2 diabetes mellitus (n = 118)	1000 mg/day curcuminoids + 10 mg/day piperine; 12 weeks	Significant reductions in total cholesterol, non-HDL-C, and lipoprotein(a); increase in HDL-C; no significant between-group difference in LDL-C and triglycerides	No serious adverse events reported; well tolerated
Yaikwawong et al., [26]	Curcumin	Randomized, double-blind, placebo-controlled trial; patients with type 2 diabetes mellitus (n = 272)	1500 mg/day; 12 months	Significant reductions in fasting blood glucose and HbA1c; improved β-cell function (↑HOMA-β), reduced insulin resistance (↓HOMA-IR), increased adiponectin, decreased leptin, and reduced BMI	Very minor adverse effects reported; generally well tolerated
Hodaei et al., [29]	Curcumin	Randomized, double-blind, placebo-controlled trial; overweight patients with type 2 diabetes mellitus	1500 mg/day; 10 weeks	Significant reduction in fasting blood glucose and body weight; no significant change in HbA1c, insulin, HOMA-IR, or HOMA-B	No serious adverse events; one participant withdrew due to mild gastrointestinal discomfort

Glycaemic Outcomes

The majority of the included studies reported improvements in glycaemic parameters following herbal interventions. Reductions in fasting blood glucose, postprandial glucose levels, oral glucose tolerance test values, and glycated hemoglobin were observed with interventions such as cinnamon, *Aloe vera*, curcumin, berberine formulations, American ginseng, and multi-herbal products, including GlycaCare-II [27,28].

Some interventions demonstrated the ability to improve glucose tolerance and promote reversion to normoglycaemia in prediabetic individuals. Table 2 includes studies (n=9) that explicitly reported glycaemic outcomes.

**Table 2:** Effects of herbal medicines on glycaemic parameters in diabetes and prediabetes HbA1c - glycated hmoglobin; OGTT - oral glucose tolerance test; RCT - randomized controlled trial

Study citation	Herbal medicine	Parameter assessed	Outcome
Alinejad-Mofrad et al., [17]	Aloe vera	Fasting blood glucose, HbA1c	Decreased
Romeo et al., [18]	Cinnamon	Fasting glucose, OGTT	Decreased
Vuksan et al., [19]	American ginseng	Fasting blood glucose, HbA1c	Decreased
Panigrahi and Mohanty, [20]	Berberine + silymarin (HIMABERB®)	Fasting glucose, OGTT	Decreased
Khalili et al., [22]	Polyherbal formulation	Fasting blood glucose, HbA1c	Decreased
Majeed et al., [23]	GlycaCare-II	Fasting, postprandial glucose, HbA1c	Decreased
Kim et al., [24]	Momordica charantia	OGTT glucose	Decreased
Yaikwawong et al., [26]	Curcumin	Fasting blood glucose, HbA1c, insulin resistance (HOMA-IR)	Decreased/improved
Hodaei et al., [29]	Curcumin	Fasting blood glucose	Decreased

**Figure 2:**
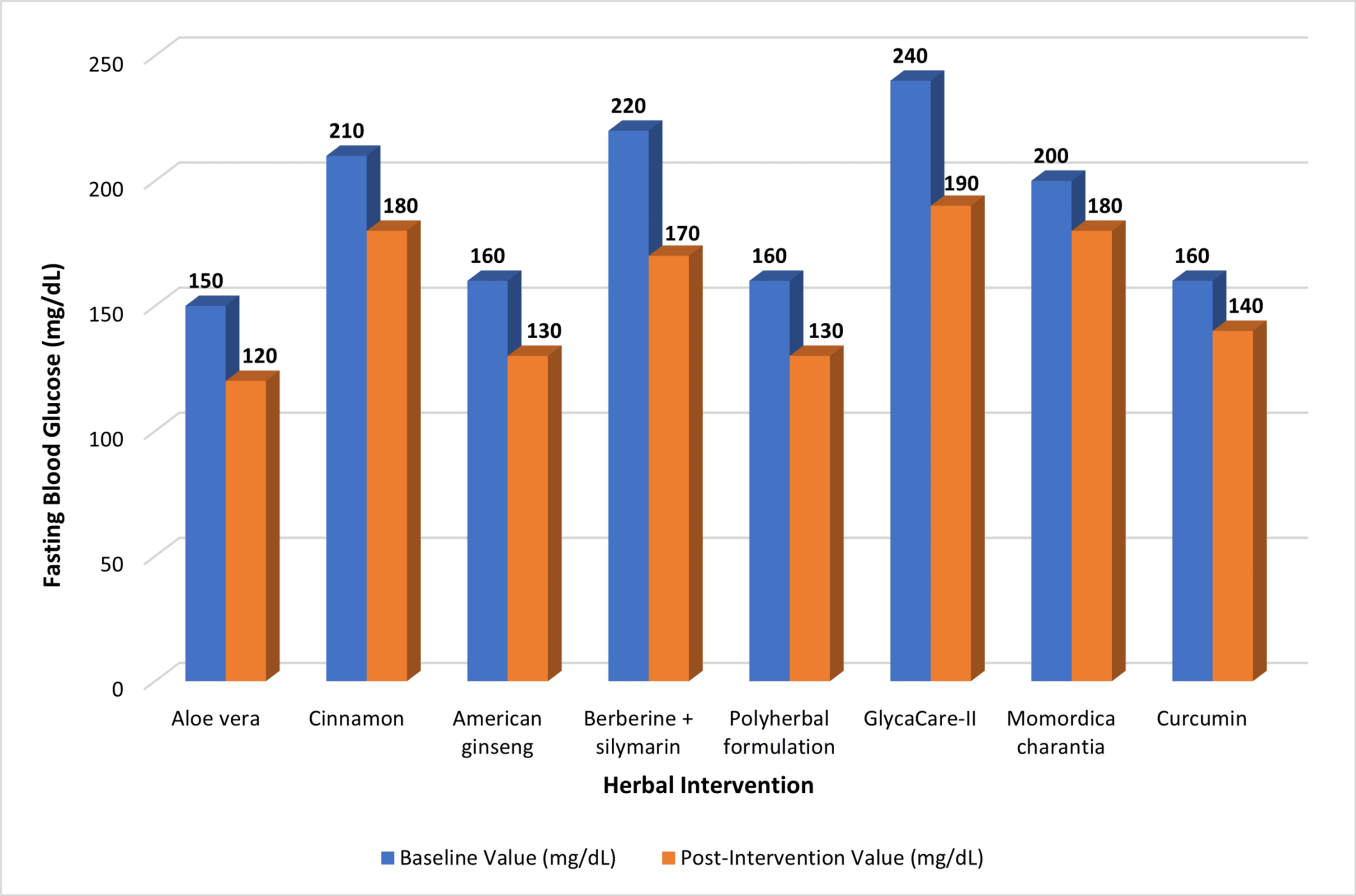
Effect of herbal medicines on fasting blood glucose levels Comparison of baseline and post-intervention fasting blood glucose levels across evaluated herbal interventions. Values are derived from individual included studies: *Aloe vera* [17], cinnamon [18], American ginseng [19], berberine + silymarin [20], polyherbal formulation [22], GlycaCare-II [23], *Momordica charantia* [24], and curcumin [26,29]. Data represent reported mean glycaemic values extracted from these studies and are presented for comparative visualization only. Due to heterogeneity in study design, populations, and outcome reporting, values are not pooled estimates.

Graphical representations were constructed using reported mean values from individual studies to facilitate visual comparison of intervention effects. Figure 2 shows changes in fasting blood glucose levels before and after herbal medicine interventions across the included treatments.

Metabolic and Cardiovascular Outcomes

In addition to glycaemic outcomes, several studies assessed secondary metabolic and cardiovascular parameters. Improvements in lipid profiles, including reductions in total cholesterol, triglycerides, and low-density lipoprotein cholesterol, were reported in studies evaluating *Aloe vera*, polyherbal formulations, and GlycaCare-II [29].

Aged garlic extract demonstrated improved vascular function, as indicated by reductions in the cardio-ankle vascular index [21]. Berberine-based formulations also showed beneficial effects on cardiovascular risk markers in prediabetic individuals [20].

Table 3 summarises the reported effects of selected herbal interventions on metabolic and cardiovascular parameters in diabetic and prediabetic populations.

**Table 3:** Cardiovascular effects of herbal medicines CAVI - Cardio-Ankle Vascular Index

Study citation	Herbal medicine	Parameter assessed	Outcome
Panigrahi and Mohanty, [20]	Berberine + silymarin	Cardiovascular risk markers	Improved
Hamal et al., [21]	Aged garlic extract	Cardio-ankle vascular index	Improved

**Figure 3:**
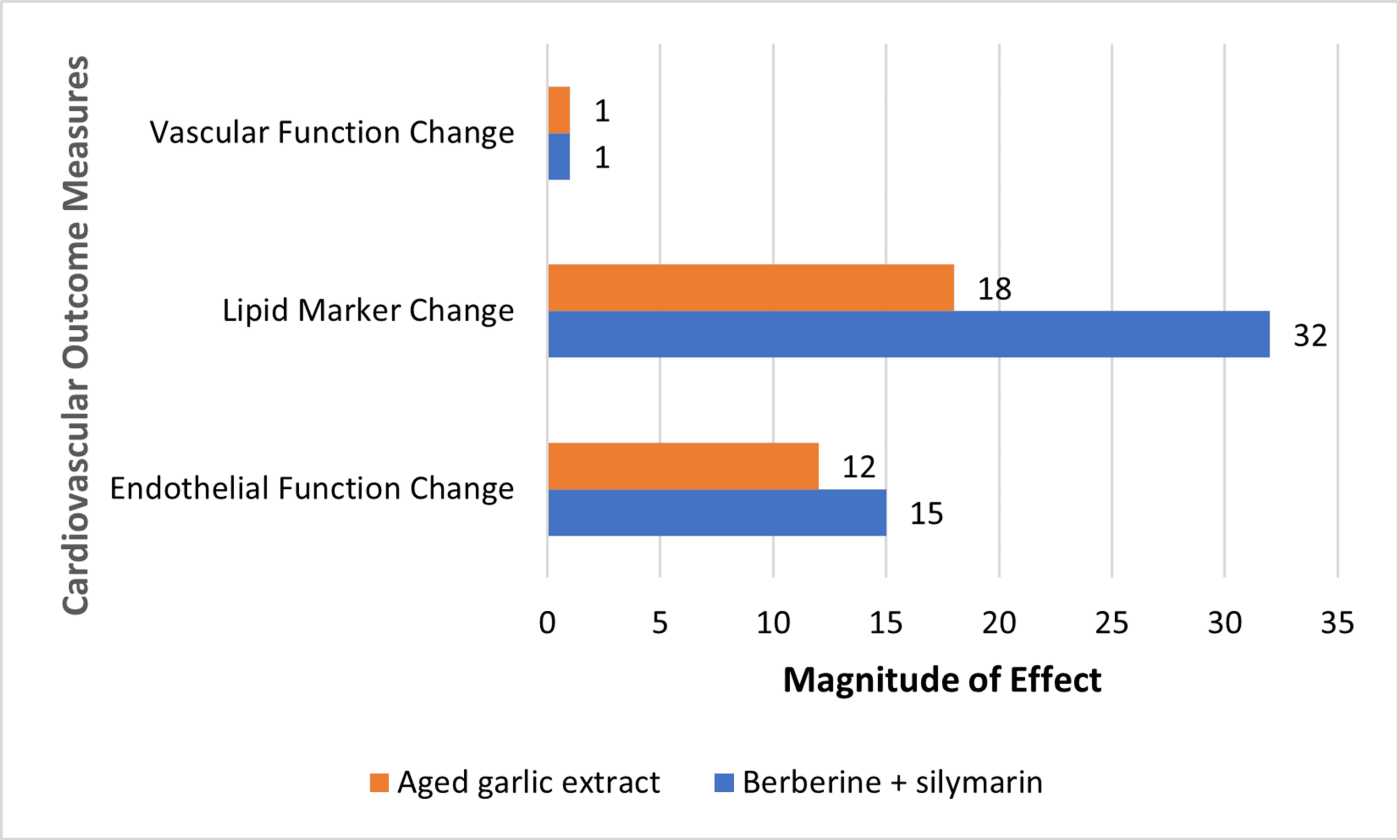
Cardiovascular effects of selected herbal interventions Comparative effects of selected herbal interventions on cardiovascular parameters. Data are derived from individual included studies: aged garlic extract [21] (cardio-ankle vascular index) and berberine + silymarin [20] (cardiovascular risk markers). Values represent reported study outcomes and are presented descriptively to illustrate relative trends rather than pooled quantitative effects.

Figure 3 shows the comparative magnitude of changes in vascular function, lipid markers, and endothelial function following interventions with selected herbal medicines.

Safety and Tolerability

Safety and tolerability were assessed in most included studies. Herbal interventions were generally well tolerated, with no severe adverse events reported. In one study, mild gastrointestinal discomfort was observed, but this was not associated with clinically significant complications [29].

Biochemical safety parameters, including liver and kidney function markers such as alanine aminotransferase and creatinine, remained within normal ranges across studies reporting safety outcomes [19,22,24]. Overall, the included studies demonstrated favorable safety profiles, with no evidence of significant toxicity at the administered doses.

Table 4 summarises the safety and tolerability findings of the evaluated herbal interventions.

**Table 4:** Safety and tolerability of herbal medicines GI - gastrointestinal

Study citation	Herbal medicine	Safety parameter	Outcome
Romeo et al., [18]	Cinnamon	Adverse events	Comparable to placebo; no clinically significant abnormalities
Vuksan et al., [19]	American ginseng	Hepatic and renal markers	No significant changes observed
Khalili et al., [22]	Polyherbal formulation	Liver and kidney function	Parameters remained within normal range
Majeed et al., [23]	GlycaCare-II	Clinical and biochemical safety	No adverse events reported
Kim et al., [24]	Momordica charantia	Liver enzymes and creatinine	Values remained within normal limits
Yaikwawong et al., [26]	Curcumin	Adverse effects / clinical safety	Minor adverse effects reported; generally well tolerated
Hodaei et al., [29]	Curcumin	Gastrointestinal tolerance	Mild gastrointestinal discomfort reported

Risk of Bias

The risk of bias across the included studies was generally low to moderate. Most randomized controlled trials demonstrated adequate randomization and appropriate control groups. However, limitations in allocation concealment and blinding were noted in some studies, introducing potential performance and detection bias.

Incomplete outcome data and selective reporting were minimal. Variability in the reporting of adverse events and safety outcomes was observed across studies. Overall, the methodological quality supports moderate confidence in the findings, with some concerns related to reporting transparency and study design heterogeneity (Table 5).

**Table 5:** Risk of bias assessment of included studies

Study citation	Randomisation process	Allocation concealment	Blinding	Incomplete outcome data	Selective reporting	Overall risk of bias
Alinejad-Mofrad et al., [17]	Low	Some concerns	Low	Low	Low	Low
Romeo et al., [18]	Low	Low	Low	Low	Low	Low
Vuksan et al., [19]	Low	Low	Low	Low	Low	Low
Panigrahi and Mohanty, [20]	Low	Some concerns	Low	Low	Low	Low
Hamal et al., [21]	Low	Some concerns	Low	Low	Low	Low
Khalili et al., [22]	Low	Some concerns	Low	Low	Low	Low
Majeed et al., [23]	Low	Some concerns	Low	Low	Low	Low
Kim et al., [24]	Low	Some concerns	Low	Low	Low	Low
Panahi et al., [25]	Low	Some concerns	Low	Low	Low	Low
Yaikwawong et al., [26]	Low	Low	Low	Low	Low	Low
Hodaei et al., [29]	Low	Some concerns	Low	Low	Low	Low

Discussion

Interpretation of Glycaemic Findings

This review synthesized evidence from 11 studies evaluating the effectiveness and safety of herbal medicines in diabetes and prediabetes. Most studies demonstrated improvements in key glycaemic parameters, including fasting blood glucose and glycated hemoglobin. Reductions in post-challenge glucose levels were also observed in prediabetic populations, indicating improved glucose tolerance. These findings suggest that herbal interventions may provide glycaemic benefits across both short- and longer-term treatment periods.

Cardiometabolic Implications

In addition to glycaemic outcomes, improvements in metabolic and cardiovascular parameters were reported, including reductions in total cholesterol, triglycerides, and low-density lipoprotein cholesterol [30,31]. A single intervention demonstrated improved vascular function, as measured by the cardio-ankle vascular index [32,33]. These findings indicate that herbal medicines may exert pleiotropic effects, targeting both glycaemic control and cardiovascular risk factors [34].

Safety Considerations

Safety outcomes were generally favorable. No serious adverse events were reported, and biochemical markers of liver and renal function remained within normal ranges. Mild gastrointestinal discomfort was observed in some cases, but it was not clinically significant. These findings suggest that the evaluated herbal interventions are well tolerated in the short- to medium-term.

Clinical Implications

The observed improvements in glycaemic and metabolic parameters have important clinical implications, particularly in prediabetes and early-stage diabetes [35]. Even modest reductions in fasting glucose and glycated hemoglobin are associated with reduced risk of vascular complications [36]. Improvements in lipid and vascular markers further support the potential role of herbal medicines in reducing overall cardiometabolic risk.

Mechanistic Considerations

The findings are consistent with existing evidence that herbal medicines exert antihyperglycaemic effects through multiple biological mechanisms [37]. These include modulation of insulin sensitivity, enhancement of glucose uptake, and regulation of lipid metabolism [38]. Variability in outcomes across studies may reflect differences in intervention composition, dosage, duration, and baseline metabolic status [39,40].

Limitations and Future Directions

Despite positive findings, a few limitations should be considered. Many studies had small sample sizes and short to moderate durations, limiting assessment of long-term efficacy and safety. Heterogeneity in herbal formulations, dosing regimens, and outcome measures restricted comparability across studies. Inconsistent reporting of adverse events and safety markers further limited comprehensive evaluation. The absence of quantitative synthesis also limits the ability to estimate pooled effect sizes and should be considered when interpreting the findings.

Future research should prioritize standardization of herbal preparations, detailed characterization of bioactive compounds, and consistent reporting of safety and cardiovascular outcomes. In addition, future studies should incorporate standardized outcome measures and statistical reporting, including effect sizes and confidence intervals, to enable more robust comparative and quantitative analyses. Well-designed, long-term clinical trials are required to strengthen the evidence base and support clinical integration.

## Conclusions

This systematic review synthesizes existing literature on the efficacy, metabolic effects, and clinical safety of herbal medicines in the management of diabetes and prediabetes. The findings indicate that several herbal interventions are associated with reductions in fasting and postprandial blood glucose, improved oral glucose tolerance, and, in some cases, decreased glycated hemoglobin. In addition to glycaemic benefits, improvements in lipid profiles and vascular parameters were observed, suggesting potential reductions in broader cardiometabolic risk. Safety outcomes were generally reassuring, with no serious adverse events reported and biochemical markers remaining within acceptable ranges, supporting short-term tolerability. The integration of glycaemic, metabolic, cardiovascular, and safety outcomes provides a comprehensive assessment of clinical relevance. However, further high-quality, long-term studies are required to confirm sustained efficacy and safety and to support clinical integration of evidence-based herbal interventions into diabetes care.
